# Brief communication: Long-term absence of Langerhans cells alters the gene expression profile of keratinocytes and dendritic epidermal T cells

**DOI:** 10.1371/journal.pone.0223397

**Published:** 2020-01-10

**Authors:** Qingtai Su, Aurélie Bouteau, Jacob Cardenas, Balaji Uthra, Yuanyaun Wang, Cynthia Smitherman, Jinghua Gu, Botond Z. Igyártó

**Affiliations:** 1 Baylor Scott & White Research Institute, Baylor Institute for Immunology Research, Dallas, Texas, United States of America; 2 Baylor University, Institute of Biomedical Studies, Waco, Texas, United States of America; 3 Baylor Scott & White Research Institute, Dallas, Texas, United States of America; 4 Thomas Jefferson University, Department of Microbiology and Immunology, Philadelphia, Pennsylvania, United States of America; Northwestern University, UNITED STATES

## Abstract

Tissue-resident and infiltrating immune cells are continuously exposed to molecules derived from the local cells that often come in form of secreted factors, such as cytokines. These factors are known to impact the immune cells’ biology. However, very little is known about whether the tissue resident immune cells in return also affect the local environment. In this study, with the help of RNA-sequencing, we show for the first time that long-term absence of epidermal resident Langerhans cells led to significant gene expression changes in the local keratinocytes and resident dendritic epidermal T cells. Thus, immune cells might play an active role in maintaining tissue homeostasis, which should be taken in consideration at data interpretation.

## Introduction

The effect of tissue environment on immune cells has been widely studied. Tissue microenvironment through an unknown mechanism is capable of shaping the chromatin landscapes of macrophages, which results in tissue-specific functions of macrophages[1]. Dendritic cell (DC) populations in different tissues display tissue-specific diversity and functions[2], and thus, it is anticipated that the close communication between DCs and the tissue microenvironment might endow them with functional diversity and plasticity. It is well documented that keratinocytes (KCs) for example can regulate immune responses by affecting epidermal resident, antigen presenting Langerhans cells’ (LCs) biology through secretion of cytokines and other factors[3]. LCs are a subset of DCs that are radiation-resistant and reside in the epidermis, where they are tightly attached to the surrounding keratinocytes[4]. LCs participate in promotion of self-tolerance, anti-fungal immunity, skin immunosurveillance, and protective humoral immune responses[5]. In this study, we tested the idea whether long-term absence of an immune cell, LCs from the epithelial environment, affects the constituent KCs and the resident dendritic epidermal T cells (DETCs). Here we show, to our knowledge, first-time evidence that long-term absence of an immune cell can lead to significant changes in the local cells and to an altered tissue microenvironment.

## Materials and methods

### Mice

huLangerin-DTA (LC^-/*-*^) mice have been previously described[6]. All experiments were performed with 8 weeks old littermate-controlled mice. Mice were housed in microisolator cages and fed autoclaved food. The Baylor Institutional Care and Use Committee specifically approved this study.

### Flow cytometry and cell sorting

Single-cell suspensions of flank skin were obtained and stained as previously described[7]. Briefly, mice were sprayed with 70% ethanol and the flank skin area shaved using Personna razor blades. The shaved area was harvested, the subcutaneous fat scraped away using forceps and ~1 cm wide strips were prepared using razor blades. The resulting skin strips were floated on Trypsin-GNK solution for ~1.5 hours in CO_2_ incubators. After the incubation the epidermal sheets were separated from the dermis using forceps and further incubated in DNase solution for 15 minutes in water bath with occasional vortexing. The resulting cell suspensions were then filtered through 40 μm cell strainers, washed and stained for flow cytometer. Cell suspensions were directly labeled with fluorochrome-conjugated antibodies for cell surface markers: MHC-II, CD45, langerin, c-kit and fixable Viability Dye. KCs (live/singlets, MHC-II^-^, CD45^-^, langerin^-^, c-kit^-^) and DETCs (live/singlets, MHC-II^-^, CD45^+^, langerin^-^, c-kit^-^) were sorted on flow cytometer (S1 Fig)[8]. Stringent doublets discrimination and live/dead gating were used to exclude possible contaminants and dead cells, respectively.

### RNA preparation

Total RNA was isolated from cell lysates using the RNeasy Micro Kit (Qiagen) including on-column DNase digestion. Total RNA was analyzed for quantity and quality using the RNA 6000 Pico Kit (Agilent).

### Sequencing library preparation

Poly-A enriched Next-Generation Sequencing (NGS) library construction was performed using the KAPA mRNA Hyper Prep Kit (KAPA Biosystems) using 50ng of input total RNA according to manufacturer’s protocol using 16 amplification cycles. Quality of the individual libraries was assessed using the High Sensitivity DNA Kit (Agilent). Individual libraries were quantitated via qPCR using the KAPA Library Quantification Kit, Universal (KAPA Biosystems) and equimolar pooled. Final pooled libraries were sequenced on an Illumina NextSeq 500 with paired-end 75 base read lengths.

### Bioinformatics analysis

Raw sequencing reads assessed for quality using FastQC software[9]. The adapters were trimmed and low-quality reads (< 20) were filtered using cutadapt[10]. Reads were aligned to the mouse reference genome (GRCm38) using hisat2. Aligned SAM (Sequence Alignment Map) files were converted to BAM (Binary Alignment Map) format using samtools[11] and featureCounts[12] was used to quantify total number of counts for each gene.

### RNA-seq analysis

Transcripts with low expression, i.e., count-per-million (CPM) > 1 in less than two samples, were removed from downstream analysis, leaving 14,964 transcripts. Differential gene expression (DGE) analysis was performed using DESeq2[13] and comparisons were made between LC^-/-^ and WT within DETC and KC cell populations.

### Pathway and gene ontology analysis

Two approaches to pathway and Gene Ontology (GO) analysis were used[14]. The Database for Annotation, Visualization and Integrated Discovery (DAVID)[15] was used for functional annotation of significantly regulated genes based on false discovery rate (FDR) < .05 and fold change (FC) cut-off of 1.5 for each comparison. Additionally, a fast implementation of pre-ranked Gene Set Enrichment Analysis (FGSEA) using the fgsea R package[16,17] was performed on KEGG (Kyoto Encyclopedia of Genes and Genomes) and GO gene sets obtained from the Molecular Signatures Database v6.2 (MSigDB)[18].

### RNA-seq data visualization

Counts were normalized using the median-of-ratios method[19] and log2 transformed for data visualization. Principal component analysis (PCA) and hierarchical clustering were performed using the R. The transcripts of all heatmaps were hierarchically clustered using Euclidean distance and complete linkage function. Heatmaps were plotted using the NMF (Non-negative Matrix Factorization) package[20], while PCA and volcano plots were made using ggplot2[21].

## Results

### Long-term absence of LCs leads to gene expression changes in KCs and DETCs

**Fig 1:**
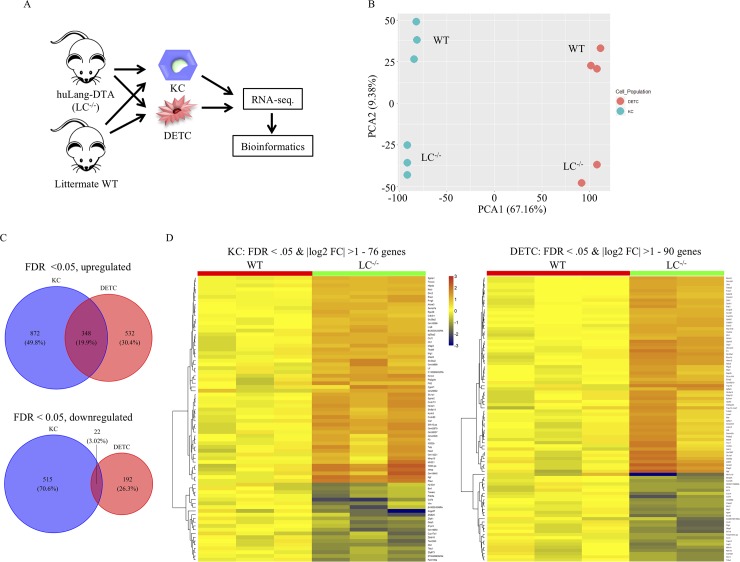
Absence of LCs leads to gene expression changes in KCs and DETCs. (A). Experimental flow. KCs and DETCs were flow sorted from LC-deficient (LC^-/-^) and littermate WT controls and RNA-seq. performed. The resulting data were then subjected to bioinformatic analyses. (B). Principal component analysis of the RNA-seq. data. Each dot represents a separate animal. (C). The overlaps between the genes that were up- (top) or downregulated (bottom) in the absence of LCs in KCs and DETCs are presented in forms of Venn diagrams; FDR<0.05. (D). Heatmap presentation of the genes that showed two-fold changes between LC^-/-^ and WT mice. KCs (left) and DETC (right). FDR<0.05.

To determine the possible effect of the absence of LCs on the cells of the epidermis, we took advantage of the huLangerin-DTA (diphtheria toxin subunit A) mice (hereafter LC^-/-^), which lack LCs starting from birth[6]. Thus, for these mice, KCs and DETCs develop, differentiate, and function in the absence of differentiated LCs. Epidermal cells suspensions were generated from a cohort of LC^-/-^ mice, along with littermate wild type (WT) controls (Fig 1A). After staining with specific markers, the KCs and DETCs were sorted using flow cytometer (S1 Fig) and RNA-sequencing performed. Unsupervised PCA of the expression data revealed that KCs and DETCs, which developed in the absence of LCs, clearly clustered away from their WT counterparts (Fig 1B). We identified 1220 up- and 537 downregulated genes in KCs, while in DETCs, we identified 880 up- and 214 downregulated genes using a false discovery rate (FDR) <0.05 (Fig 1C). Out of the upregulated genes, 348 (19.9%) were common between KCs and DETCs, while 22 genes (3.02%) were commonly downregulated (Fig 1C). Next, we performed hierarchical clustering of differentially expressed genes with at least 2-fold change and plotted heatmaps to show the distinct patterns of up- and downregulated genes in KCs and DETCs (Fig 1D).

**Fig 2:**
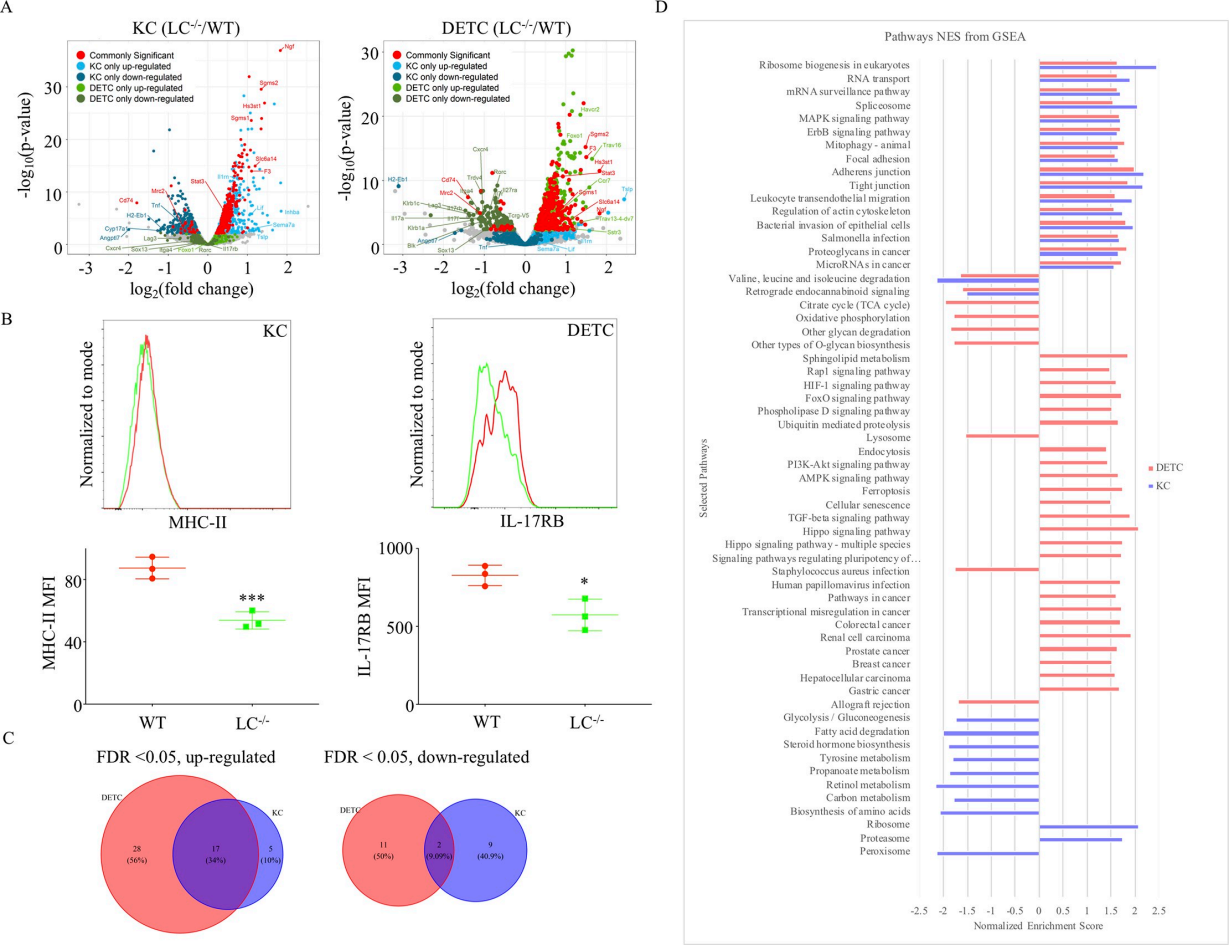
LCs have common and cell specific effects on KCs’ and DETCs’ biology. (A). Common and cell specific gene expression changes are presented in form of color-coded volcano plots. KCs (left) and DETCs (right). (B). Flow cytometry confirmation of the RNA-seq. data on protein levels. Each dot represents a separate mouse. Two tailed Student’s t-test. *p<0.05, ***p<0.001. (C). The overlap between up- or downregulated regulated KEGG pathways in KCs and DETCs from LC^-/-^ mice are presented in forms of Venn diagrams. (D). Selected KEGG pathways altered in KCs and DETCs in the absence of LCs are depicted based on normalized enrichment scores (NES). FDR<0.05.

We used color-coded volcano plots to better capture and visualize the common and cell specific changes in gene expression (Fig 2A; for DGEs please see S1 and S2 Files). We observed that in the absence of LCs nerve growth factor (*Ngf*) was the most highly upregulated gene in both KCs and DETCs. NGF is part of the neurotrophin family and is involved in the differentiation and survival of neuronal cells[22], which suggest that LCs might directly or indirectly regulate nerve homeostasis in the epidermis[23].

Out of highly published, skin-relevant molecules, thymic stromal lymphopoietin *(Tslp)* was upregulated in the KCs (trend in DETCs) in the absence of LCs, which confirms the findings recently published by Lee et al.[24]. TSLP is a known regulator of the Th2 responses and it is also needed for mast cell homeostasis[25,26]. Dysregulated TSLP production by KCs, in the absence of LCs, could have contributed to altered IgE levels[27] and increased mast cell numbers observed in the LC^-/-^ mice (FVB background). The KCs, among others, showed downregulation of the MHC-II pathway genes *H2-Eb1* (Fig 2A and 2B), *Cd74* (invariant chain; also downregulated in DETCs;), and *Cyp17a1*, a member of the P450 cytochrome family involved in carcinogen metabolism[28,29]. MHC-II and CD74 are highly expressed in LCs, and in light of our recent observation that LCs is involved in bidirectional mRNA exchange with KCs and DETCs[8], it is plausible that the observed decrease of the MHC-II pathway genes in KCs and DETCs might be due to the absence of LCs as a source of these mRNAs rather than changes in transcription.

### Lack of LCs might affect DETCs’ biology and homeostasis

We discovered that DETCs downregulated the Th17 pathway associated molecules, including *Rorc* transcription factor, *Il17rb* receptor, and *Il17a* and *Il17f* cytokines (Fig 2A and 2B). The IL-17 pathway plays an important role in the DETC’s innate immune function to fight bacterial infections[30]. More interestingly, we observed that DETCs showed lower expression of the γ/δ TCRs (*Trdv4* and *Tcrg-V5*) and upregulation of TCR alpha chains (*Trav16* and *Trav13-4-dv7*). Transcription factors and other molecules that regulate the development, differentiation, and homeostasis of DETCs, such as *Sox13*, *Blk*, and *Il7r*[31], were also downregulated. Thus, these data suggest that LCs might directly or indirectly regulate DETCs’ biology and homeostasis, and could contribute to maintain their identity/fitness in the epidermis/periphery in the absence of the thymic environment. Contrary to our findings, a previous report presented data supporting that LCs are not required for DETCs’ homeostasis and function[32]. In this manuscript the authors focused on whether the absence of LCs affects DETC’s density, steady state and activation markers, cytotoxic activity and reaction to skin injury. The manuscript reported no changes in the limited number of markers studied nor defect in DETCs’ cytotoxic activity. Our data confirm their findings on mRNA levels (please see DGE supplementary data). However, they did report a significant increase in the size of activated cell region in the LC deficient mice 48 hours post skin injury. Thus, these data collectively support that LCs can specifically affect certain aspects of DETC’s biology.

### The absence of LCs affected a variety of different cellular pathways in KCs and DETCs

To gain a broader picture about the effect of the absence of LCs on KCs and DETCs, we performed KEGG pathway analysis on the expression data. We present data of significantly altered pathways using FDR < 0.05. We observed significant overlap of pathways upregulated by KCs and DETCs, but very minimal overlap of downregulated pathways (Fig 2C). The commonly upregulated pathways included different forms of cell adhesions (focal, adherent and tight)-, ribosome and RNA biogenesis-, autophagy-, bacterial invasion/infection-, MAPK- and ErbB signaling pathways (Fig 2D). Alterations in adhesion molecules and the ErbB signaling pathway in KCs, in the absence of LCs, were recently reported[24,33]. The downregulated pathways showed considerably less overlap between these two cell types and included some of the amino acid degradation pathways (Fig 2D). KCs showed distinct dysregulation (mostly downregulation) of various metabolic pathways (sugar, protein, fatty acids, hormones, drug, xenobiotics etc.), while DETCs presented with alterations in TGF-β-, Hippo-, oxidative phosphorylation-, citrate cycle-, lipid metabolism-, Staphylococcus aureus infection- etc. pathways (Fig 2D). Thus, these data suggest that LCs might have common and cell-specific effects on KCs’ and DETCs’ biology.

## Discussion

Here we bring experimental evidence that long-term absence of LCs leads to gene expression changes in KCs and DETCs. The significant changes discovered by pathway analysis also suggest that KCs’ and DETCs’ biology and hemostasis are likely affected. Further studies are indeed needed to confirm the observed changes and their consequences. It will also be important to determine which LC-derived factors play role in the epidermal homeostasis. Our preliminary IPA Upstream Regulator Analysis identified a list of potential regulators, including cytokines, growth factors, membrane proteins and enzymes (please see S3 and S4 Files), known or anticipated to be expressed by LCs.

It will be critical to determine whether acute depletion of LCs would lead to similar changes in the local environment, and if yes, how long the LCs have to be depleted for, before their absence is noticed. These studies probably should be carried out side-by-side using co-housed (or intercrossed) huLangerin-DTR and muLangerin-DTR mice treated or not with DT. Performing the experiments this way, should help to minimalize the possible effect of colony-related microbiome differences, that alone, could lead to gene expression changes in the local cells. Furthermore, these experiments could also help to reconcile the discrepancies reported -especially fueled by a single study[34]- using these two inducible systems[35]. Reconstitution of irradiated LC^-/-^ mice with WT bone marrow or hu/muLangerin-DTR injected with DT and left to recover the depleted LCs, could also test whether the repopulating LCs can substitute for yolk sac/fetal liver-derived LCs[36] and normalize the epidermal homeostasis.

To our knowledge we show for the first time that long-term absence of an immune cell can lead to significant changes in the local cells and to altered tissue environment. The effect of local cells on resident immune cells is very much appreciated by the immunologist, however, our findings support the idea that the resident immune cells are not mere passive receivers, but rather play an active and indispensable role in maintaining tissue homeostasis. It is expected that our findings will not be limited to LCs or LC-derived factors, and that the long-term absence of other DCs, DC molecules or immune cells would lead to specific changes in the local environment. Thus, studies using constitutive immune-cell knockouts, including LC^-/-^ mice, in which the immunological changes and outcomes were directly attributed to the absence of a specific immune cell, might have to be reassessed[5,6,37–39].

## Supporting information

S1 Fig Gating strategy for sorting. (PDF)

S1 File KC_DTA_vs_WT_deseq2_results. (CSV)

S2 File DETC_DTA_vs_WT_deseq2_results. (CSV)

S3 File KC_Upstream_regulators. (XLS)

S4 File DETC_Upstream_regulators. (XLS)
