# Supplementary figures and images for: Brief communication: Long-term absence of Langerhans cells alters the gene expression profile of keratinocytes and dendritic epidermal T cells
Source: PLoS One. 2020 Jan 10;15(1):e0223397. doi: 10.1371/journal.pone.0223397 (PMC6953782; doi:10.1371/journal.pone.0223397)

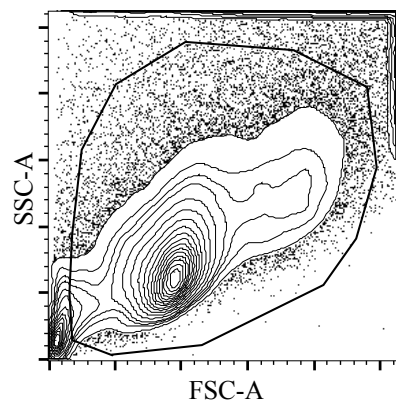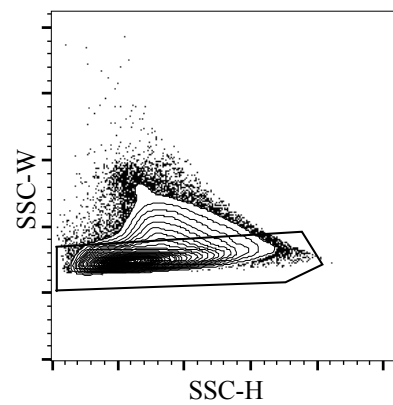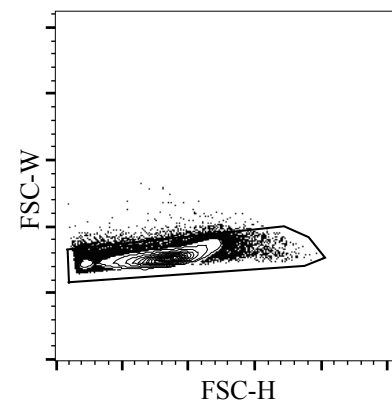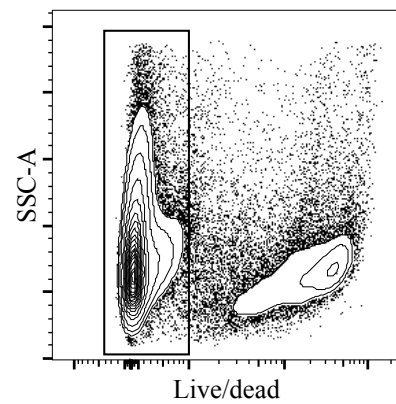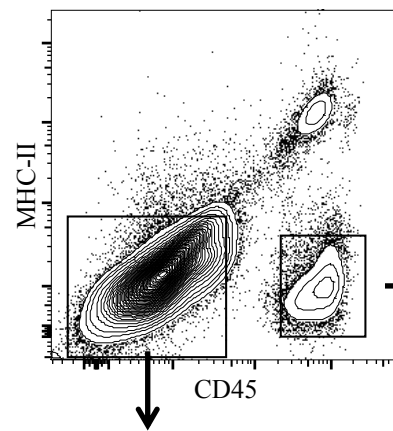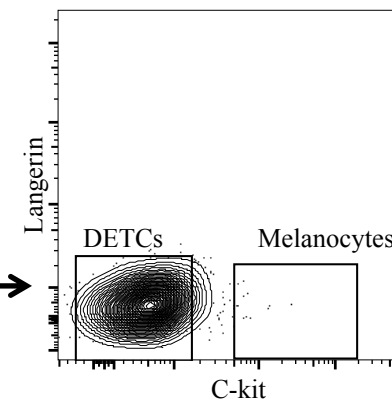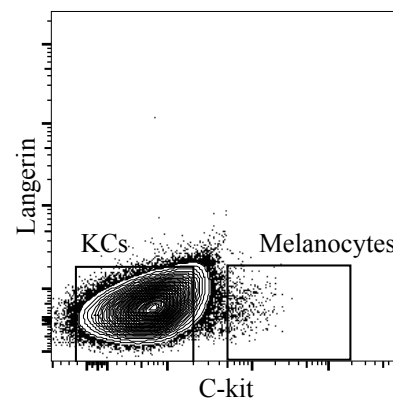

Supplement: S1 Fig — (PDF) [file pone.0223397.s001.pdf]
